# Supplementary material for: Simulations of magnetization reversal in FM/AFM bilayers with THz frequency pulses
Source: Sci Rep. 2023 Jul 28;13:12270. doi: 10.1038/s41598-023-39175-6 (PMC10382508; doi:10.1038/s41598-023-39175-6)
Supplement: Supplementary file 1 — Supplementary Information. [file 41598_2023_39175_MOESM1_ESM.pdf]

Supplementary Information: Simulations of Magnetisation Reversal in FM/AFM Bilayers With THz Frequency Pulses

Joel Hirst[1*], Sergiu Ruta[1,2], Jerome Jackson[3], Thomas Ostler[4]

[1]Materials & Engineering Research Institute, Sheffield Hallam University, Howard Street, Sheffield, S1 1WB, United Kingdom.
[2]The Department of Engineering and Mathematics, Sheffield Hallam University, Howard Street, Sheffield, S1 1WB, United Kingdom.
[3]Scientific Computing Department, STFC Daresbury Laboratory, , Warrington, WA4 4AD, United Kingdom.
[4]Department of Physics & Mathematics, University of Hull, , Hull, HU6 7RX, United Kingdom.

*Corresponding author(s). E-mail(s): joel.hirst@student.shu.ac.uk;

S1: Derivation of Torquance

The staggered SO field profile used in the atomistic simulations is given by the equation:

$$B(t) = H \exp\left(-\frac{(t-t_0)^2}{2\sigma^2}\right) \sin\left(2\pi f\left(t-t_0\right)\right) \tag{1}$$

Where H is a prefactor used to vary the field strength, σ is the standard deviation, t_0 is the central position of the pulse, and f is the frequency of the pulse. At 0 K, the anisotropy field is $H_{\mathrm{an}} = 0.174$ T. The exchange field can be related to the sum of the inter-sublattice exchange constants via $H_{\mathrm{ex}} = J_{0,\nu\kappa}/\mu_s = 1872$ T where ν and κ represent each sublattice. For the fitting to the 300 K magnetisation dynamics, we assume the cubic anisotropy scales with the $K(T) = Km_e^{10}(T)$ [1] and the exchange field scales with $m_e(T)$. To obtain a value for the NSOT torquance, λ_{NSOT}, we fit the magnetisation dynamics at both $T = 0$ and 300 K to the following differential equation:

$$\ddot{\varphi}_{\mathrm{L}} = -\gamma H_{\mathrm{ex}}\left(2\alpha_{\mathrm{G}}\dot{\varphi}_{\mathrm{L}} + \gamma H_{\mathrm{an}}\sin\left(4\varphi_{\mathrm{L}}\right) + \lambda_{\mathrm{NSOT}}\sigma_c H \exp\left(-\frac{(t-t_0)^2}{2\sigma^2}\right)\sin\left(2\pi f\left(t-t_0\right)\right)\cos\left(\varphi_{\mathrm{L}}\right)\right) \tag{2}$$

We fit the differential equation above and extract a value of $\lambda\sigma H$. By plotting the extracted value of $\lambda\sigma H$ to the actual value of H used in the atomistic simulations we get a gradient corresponding to $\lambda\sigma$. The extracted values of $\lambda\sigma H$ are shown in Supplementary Fig. 1. Red circles and blue squares are for extracted values at 0 K and 300 K respectively. The solid red line is a fit to the 0K data. A value of $\sigma = 1.5 \times 10^5$ m^{-1} V^{-1} A [2] is used for both temperatures. The value of the torquance at 0 and 300 K are almost identical with $\lambda_{\mathrm{NSOT}} = 234 \pm 4$ and $\lambda_{NSOT} = 240 \pm 12$ cm A^{-1} s^{-1} for the two temperatures respectively.

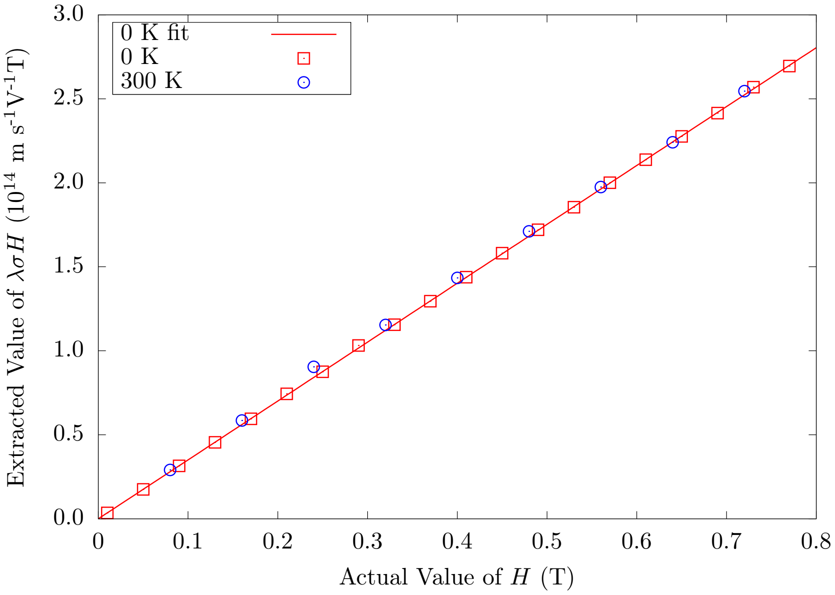

Fig. 1: Extracted values of the prefactor $\sigma\lambda_{\rm NSOT}H$. red squares and blue circles are for 0 K and 300 K respectively. Solid red line is a fit to the 0 K dynamics, from which the gradient gives the value of $\lambda_{\rm NSOT}$.

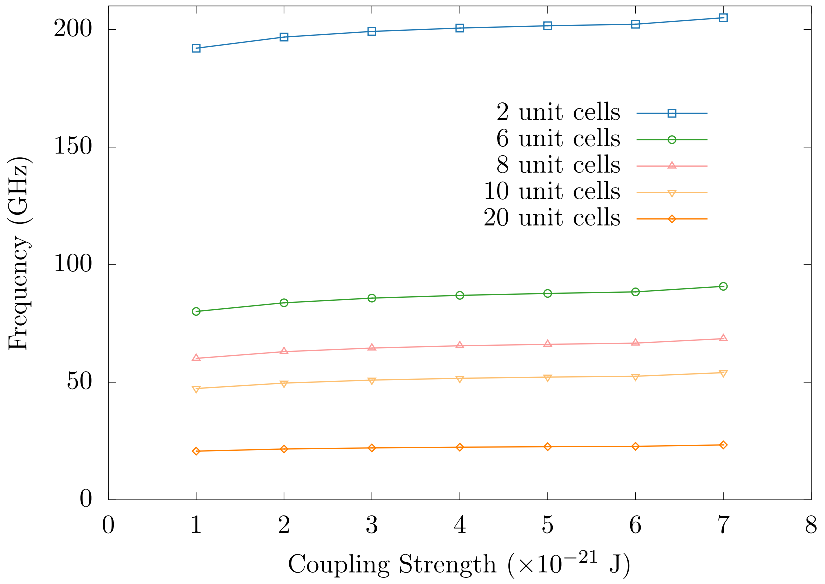

Fig. 2: The resonant frequency of the bilayer as a function of interface exchange. Individual lines show varying thickness of Permalloy.

S2: Resonant Frequency scaling of Bilayer

Both the thickness of the Permalloy and interface exchange, $J_{ij}^{\rm Inter}$, effect resonant frequency mode of the bilayer. To better quantify this, we calculate the frequency both as a function of FM thickness and interface exchange. To excite the resonant frequency mode, all spins in the chain are rotated by 20 $^\circ$ in the xy-plane and then released to induce a precession. The magnetisation dynamics are are recorded for 50 ns at $T = 0$K. The frequency is found via Fast Fourier Transform of the outputted dynamics. Fig 2 shows the frequency of the lowest (n=0) resonance mode. The results show that for thinner thicknesses of Permalloy, the higher the resonant frequency as the faster magnetisation dynamics of the AFM start to dominate. The coupling strength has a lesser impact on the frequency dynamics. A 7 fold increase from $J_{ij}^{\rm Inter} = 1.0 \times 10^{-21}$ J to 7.0×10^{-21} J yields in increase of between ~ 1.2 and 1.3 times increase in the resonance frequency.

References

[1] Asselin, P., Evans, R.F.L., Barker, J., Chantrell, R.W., Yanes, R., Chubykalo-Fesenko, O., Hinzke, D., Nowak, U.: Constrained monte carlo method and calculation of the temperature dependence of magnetic anisotropy. Phys. Rev. B **82**, 054415 (2010) https://doi.org/10.1103/PhysRevB.82.054415

[2] Gomonay, O., Jungwirth, T., Sinova, J.: Narrow-band tunable terahertz detector in antiferromagnets via staggered-field and antidamping torques. Physical Review B **98**, 1–8 (2018) https://doi.org/10.1103/PhysRevB.98.104430
